# Mapping Value Co-creation Literature in the Technology and Innovation Management Field: A Bibliographic Coupling Analysis

**DOI:** 10.3389/fpsyg.2020.588648

**Published:** 2020-09-25

**Authors:** Juan-José Nájera-Sánchez, Marta Ortiz-de-Urbina-Criado, Eva-María Mora-Valentín

**Affiliations:** Facultad de Ciencias Jurídicas y Sociales, Universidad Rey Juan Carlos, Madrid, Spain

**Keywords:** value co-creation, technology and innovation management, mapping, bibliographic coupling analysis, open innovation, servitization, sharing economy

## Abstract

Value co-creation has become a very important topic in several disciplines. It is observed that value co-creation has been analyzed mainly from a perspective of marketing or services. The interest of studying value co-creation in relation to innovation is growing but there are no previous literature reviews that focus on the literature that studies value co-creation from a technology and innovation management perspective. The present research aims to close this gap. This research has two aims. First, we make a descriptive analysis of the evolution of documents published from 2004 to 2020. We analyze the main journals and identify the most prolific authors. In addition, we observe collaborative behavior at three different levels – country, institution, and author. Second, we determine the content structure of this literature through a bibliographic coupling analysis, and characterize the resulting groups. As a result of this analysis, we describe eleven thematic groups and characterize them through different metrics. Based on these metrics and the previous analysis, we classify and explain the studies about co-creation in the technology and innovation management field. We obtained three research streams: open innovation, consumer-centric analysis, and service ecosystem and service innovation, and two new trends: servitization and the sharing economy.

## Introduction

The term co-creation was popularized in the business context by Prahalad and Ramaswamy (2000, 2004) in an attempt to gauge the dynamics of the relationship between companies and consumers (Ramaswamy and Gouillart, 2010). Research about value co-creation is in an evolutionary phase and has received major attention from academics (Bharti et al., 2015). However, several studies have emphasized the need to deepen the theoretical fundamentals of this subject (Gebauer et al., 2010; Paulin and Ferguson, 2010; Loss and Crave, 2011). According to Saha et al. (2020), it is important to systematically investigate the concept of value co-creation to gain some useful insights that would be helpful for both industry and academia. Our paper is a step in that direction.

The growth of the literature on value co-creation has stimulated the publication of reviews, using different approaches. The first approximations were generic. Galvagno and Dalli (2014) performed a co-citation analysis and they identify three main theoretical perspectives to study co-creation: services sciences, marketing and consumer research, and innovation and technology management. Bharti et al. (2015) use thematic content analysis to identify 27 elements of co-creation and classify them into five categories: process environment, resource, co-production, perceived benefits, and management structure. Leclercq et al. (2016) conduct an integrative review about value co-creation from the innovation, business, and marketing disciplines. In the following years, some other studies have complemented these first attempts, using bibliometric techniques and adopting general approaches (Alves et al., 2016; Ribeiro et al., 2016; Saha et al., 2020).

Other works have analyzed value co-creation in specific domains such as tourism (Campos et al., 2015; Tregua et al., 2020), public services (Voorberg et al., 2015; Nicola et al., 2019) or health care (Greenhalgh et al., 2016; Aghdam et al., 2020), or regarding particular issues as the value co-creation process in web-based multisided platforms (de Oliveira and Cortimiglia, 2017) or value co-creation in online communities (Priharsari et al., 2020).

Another group of literature reviews has focused on the field of technology and innovation management. Although value co-creation has been analyzed mainly from a perspective of marketing or services, technology and innovation management has been one of the main pillars for this literature (Galvagno and Dalli, 2014). In fact, several articles have reviewed specific aspects of this literature. For example, Ramírez-Montoya and García-Peñalvo (2018) make a systematic literature review about open innovation and the co-creation of knowledge to promote open science. Tekic and Willoughby (2019) conduct a systematic review of the innovation management literature for clarifying the concepts of co-creation and open innovation using bibliometric analysis.

The interest in studying value co-creation in the context of technology and innovation literature is growing. There are several roles that a customer can play in the innovation process: as an information source, co-developer, and innovator (Cui and Wu, 2017). All of these roles have been analyzed in recent years, because of their potential to affect firms’ competitive advantage. Also, the phenomenon of value co-creation has extended to other stakeholders, to study their contribution to the innovation process (Akesson et al., 2016). Value co-creation related to technology and innovation management has expanded in several industries, with special intensity in services (tourism, health, and public services) but not exclusively. This heterogeneity has originated a very complex research field that requires effort to produce order and systematization. However, to the best of our knowledge, there are no previous literature reviews that focus on value co-creation from a technology and innovation management perspective. The present research aims to fill this gap by carrying out a bibliometric study to analyze the previous literature on co-creation from the perspective of technology and innovation management and, in this way, to systematize this literature. This study attempts to answer the following research questions: (1) What are the main journals and who are the most prolific authors in the field of value co-creation from a technology and innovation management perspective? What is the collaborative behavior like between countries, institutions, and authors? (2) What is the knowledge structure of the literature about value co-creation from a technology and innovation management perspective? (3) What are the emerging themes in the field of value co-creation from a technology and innovation management perspective?

To answer these questions, first, we introduce the methodological aspects, going deeper in the different phases we have followed, from delimiting our sample to analyzing the knowledge structure. After that, we make a descriptive analysis of the evolution of documents published from 2004 to 2020. We establish 2004 as the initial point because even though the seminal work is previous (Prahalad and Ramaswamy, 2000), the contributions to the field start to be published in a consistent way after 2004. With the first group of questions, we will shed light on the main characteristics of this literature, determining the most prolific authors, universities, and countries working in this topic, the pattern of collaboration among them (social structure), and the publications editing this research. The aforementioned heterogeneity drives the appearance of researchers and publications that have published works in this topic only in a tangential way. Also, we observe the collaborative behavior at three different levels – country, institution, and author.

Questions 2 and 3 deal with the knowledge structure of the field. The huge variety of subtopics and industries justifies the necessity of systematization, which can also contribute to finding underexplored research lines. In this case, we have used the bibliographic coupling technique that is based on the analysis of shared cited references among papers: if two references are sharing the same sources, they are analyzing similar topics. Based on this argument, we can group the literature in the field and analyze what are these sub-topics and how they are related. We complete this analysis with a more quantitative study of the different groups to determine their evolution and the role they play in the research field. Finally, we present some insights with respect to the future development of this line of research.

## Methodology

To accomplish our research objectives, we have followed the scheme proposed by Kovács et al. (2015), with some adaptations. We have completed four steps: (1) building the database of citing references; (2) preparing the database for analysis; (3) mapping the documents using the bibliographic coupling procedure; and (4) analyzing the networks, adding some relevant information to assess the impact of different topics, and their likely future behavior.

To build the database, we gathered the citing references from the Web of Science (WoS) Core Collection. To guarantee the quality of these documents, we only looked up papers indexed in the Social Science Citation Index (SSCI). Other studies in similar areas have taken the same decision (Teixeira and Mota, 2012; Meyer et al., 2014; Skute et al., 2019). Zupic and Čater (2015) showed this is the most common option in social sciences-related research.

Following previous reviews, we searched the title, abstract, and keywords fields (author and keyword plus) with the following query: (co-creat^∗^OR cocreat^∗^ OR “co creat^∗^”) AND (innovat^∗^ OR technol^∗^). We limited the results by year (documents published after 2004 included) and type of document (all categories excluded except articles and reviews). Using this search strategy, the query returned 1,708 documents (on May 20th, 2020).

Once we built our database, we used Bibexcel software (Persson et al., 2009) to prepare the data. One of the most highlighted problems of bibliographic coupling is the codification of cited references. All the databases (and WoS is not an exception) have inconsistencies in this field. It is easy to find the same reference written in several different ways. To avoid this, we manually checked all the references to look for inconsistencies. Also, to run all the descriptive statistics we checked author names and affiliations.

We choose bibliographic coupling (Kessler, 1963; Boyack and Klavans, 2010; Mura et al., 2018; Skute et al., 2019) to answer questions 2 and 3, related to the knowledge structure of the field and its emerging topics. Zupic and Čater (2015) point out that bibliographic coupling is an adequate approach to analyze recently published documents. That suitability is due to the focus of this technique on citing documents, using cited references to establish links among them. Some authors have defended the results of this methodology as more precise than results from co-citation analysis or citation analysis (Boyack and Klavans, 2010). In dynamic research fields, the performance of bibliographic coupling is even better, in comparison with other techniques (Vogel and Güttel, 2013).

Our level of analysis was the document. The underlying principle in this technique is that two documents citing the same references are studying similar topics or share a common perspective. In other words, they share the intellectual base (cited references).

**FIGURE 1:**
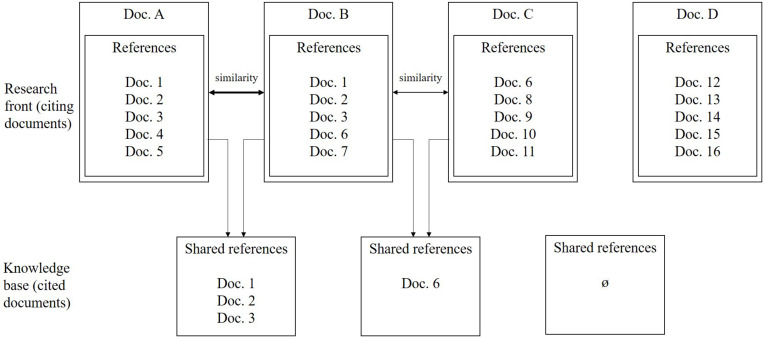
Bibliographic coupling.

Figure 1 illustrates different cases. Documents A and B have three references in common while papers B and C share just one. In both situations, there is a relationship between the citing documents, although the intensity of that link is higher between A and B (the similarity is higher). Reference D has no relationship with the other documents, because it does not share any cited references. Using social network analysis techniques, the citing documents are represented as nodes and the links between them are based on those relationships that represent similarity among documents. To avoid spurious relationships, a minimum threshold of shared references to make a coupling is established. Analyzing these similarities between documents, it is possible to group them in homogeneous thematic clusters. These clusters are usually related, although the relationships between clusters are weaker than the relationships between papers in a cluster. This technique allows us to describe the knowledge structure of a research field.

This way, we had to make three decisions to carry out the bibliographic coupling. First, we had to elect a measure of similarity. We adopted association strength (van Eck and Waltman, 2009). Second, we chose VOS as the grouping algorithm (Waltman et al., 2010; van Eck and Waltman, 2014). A lot of researchers have corroborated its good results (Kovács et al., 2015; van Oorschot et al., 2018; Skute et al., 2019). Third, to set a threshold for coupling (citations in common), we followed (Mura et al., 2018). We tested different thresholds and we set it at 18 documents. That was the best solution because of the number of clusters and their internal consistency. Additionally, to simplify the network, we set a minimum degree of two for an article to remain in the network (Vogel and Güttel, 2013). Finally, we analyzed the 131 documents included in the network.

## Results

### Descriptive Analysis

**FIGURE 2:**
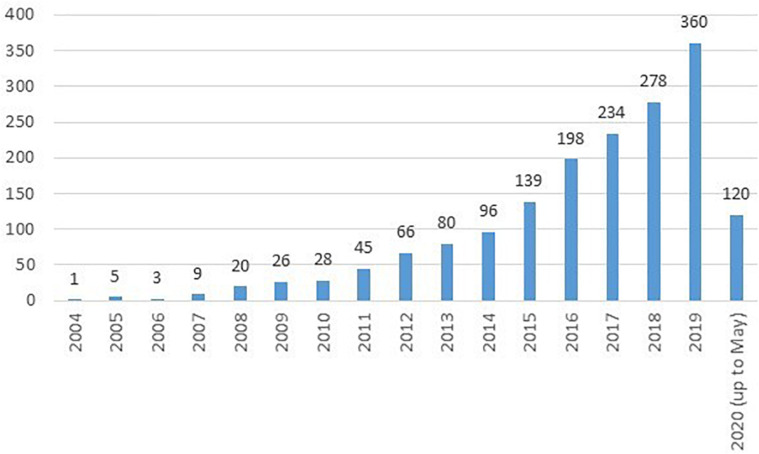
Evolution of number of published documents.

Figure 2 shows the temporal distribution of published documents since 2004. The number of documents in the period 2004–2020 (until May) was 1,708. According to the evolution of this number, we identify three phases: 2004–2009, 2010–2014, and 2015–2020. The first period (2004–2009) is very incipient, with an erratic pattern of publication. From 2010 to 2014, the number of papers grows significantly, i.e., a consolidating period. The last phase shows an exponential growth of scientific production, confirming the interest of the academic community in the value co-creation phenomenon.

Table 1 includes the 20 most frequent journals in this literature. Considering the full period (2004–2020), the top 4 journals in the ranking are Sustainability, Journal of Business Research, Industrial Marketing Management, and Journal of Service Management. These journals have published around 14% (239) of the documents in our database. We highlight the behavior of Sustainability (indexed in SSCI in recent years, our database only includes papers from this journal at the end of the period), and the Journal of Business Research (in which this topic has occupied a standout place just in the last lustrum). The very different nature of the top four journals is interesting: Sustainability aside, the Journal of Business Research is a generalist business/management publication; Industrial Marketing Management focuses on marketing-related themes; the Journal of Service Management publishes papers centered on the service industry. This heterogeneity is a distinguishing mark of value co-creation literature. In the top 20 journals, the thematic variety is noticeable, particularly the inclusion of several technology and innovation management (TIM) journals. Also, we highlight the presence of information technology (IT) publications, a fundamental facilitator of co-creation, and tourism specialized journals, an industry in which the studied phenomenon performs a leading role. Most of the journals in Table 1 have published the majority of the documents in the last 5 years (2015–2020), although some of them have behaved differently, without an evident pattern.

**TABLE 1:** Top 20 journals publishing co-creation related documents.

Journal	Last period	Full period
	
	2015–2020	2004–2020
Sustainability	67	67
Journal of Business Research	59	64
Industrial Marketing Management	45	55
Journal of Service Management	41	53
Journal of Business and Industrial Marketing	28	32
International Journal of Contemporary Hospitality Management	29	30
Journal of Cleaner Production	26	29
Technological Forecasting and Social Change	25	28
Journal of Product Innovation Management	14	26
Journal of Services Marketing	24	26
Journal of Service Research	12	21
European Journal of Marketing	14	20
Service Science	14	19
Journal of Service Theory and Practice	18	18
Service Industries Journal	14	18
International Journal of Information Management	15	16
Computers in Human Behavior	8	15
Creativity and Innovation Management	7	15
International Journal of Hospitality Management	14	15
Journal of Marketing Management	14	14

Table 2 contains the top 10 WoS categories in the value co-creation literature. We point out that a publication can be in more than one of these categories. For the 2004–2020 period, Business and Management categories are the most frequent. The rest of the categories have a much lower proportion in the database. However, the evolution shows a diminishing tendency of the proportion of documents in Business and Management categories whilst Hospitality, Leisure, Sport and Tourism, and Environmental Studies have grown.

**TABLE 2:** Top 10 WoS categories.

WoS category	Last period		Full period	
	2015–2020		2004–2020	

	Documents	%	Documents	%
Business	484	36.4%	634	37.1%
Management	463	34.8%	633	37.1%
Hospitality, leisure, sport and tourism	113	8.5%	128	7.5%
Environmental studies	118	8.9%	124	7.3%
Information science and library science	95	7.1%	123	7.2%
Environmental sciences	116	8.7%	120	7.0%
Green and sustainable science and technology	109	8.2%	113	6.6%
Engineering, industrial	58	4.4%	89	5.2%
Computer science, information systems	57	4.3%	80	4.7%
Education and educational research	49	3.7%	60	3.5%

In relation to affiliation, the United States and England gather the biggest proportion of papers of our database (Table 3), followed by Netherlands, Australia, Germany, China, and Sweden. All of these countries have more than 200 published papers between 2004 and 2020. Some of the countries stand out because of their growth, especially Australia, China, and Italy.

**TABLE 3:** Top 10 countries publishing co-creation related documents.

	Last period	Full period
	
Country	2015–2020	2004–2020
United States	548	735
England	397	478
Netherlands	187	237
Australia	208	231
Germany	176	211
Peoples R China	186	204
Sweden	160	201
Italy	167	195
Finland	159	192
Spain	128	156

Table 4 includes the institutions where authors in this topic work. Considering the full period, among the top 15 universities, there are four from The Netherlands (Maastricht University, Wageningen University, Delft University of Technology, and Erasmus University Rotterdam), several from the Nordic countries (three from Sweden, Karlstad University, Linkoping University, and Lulea University of Technology, two from Finland, Aalto University and Hanken School of Economics, and one from Denmark, Copenhagen Business School), two from England (University of Manchester and University of Nottingham) and one from Central Europe (University of Innsbruck from Austria). Only Hong Kong Polytechnic University and University of Auckland are from non-European countries. Only the University of Innsbruck exhibits a decreasing behavior. On the contrary, several universities show astonishing growth rates in published documents on the topic.

**TABLE 4:** Top 15 institutions publishing co-creation related documents.

	Last period	Full period
	
Institution (Country)	2015–2020	2004–2020
Karlstad University (Sweden)	31	51
Maastricht University (Netherlands)	29	39
Aalto University (Finland)	29	37
Linköping University (Sweden)	27	33
University of Innsbruck (Austria)	8	27
Wageningen University (Netherlands)	17	24
University of Manchester (England)	18	24
University of Nottingham (England)	19	23
Copenhagen Business School (Denmark)	15	23
Hong Kong Polytechnic University (China)	19	22
Hanken School of Economics (Finland)	20	22
University of Auckland (New Zealand)	16	21
Lulea University of Technology (Sweden)	19	21
Delft University of Technology (Netherlands)	19	21
Erasmus University Rotterdam (Netherlands)	16	20

Table 5 includes the authors who have published six or more papers contained in our database and the number of citations of the documents associated with each author in the WoS database. The first position in this ranking is for professor Edvarsson, from Karlstad University (also in the first place in the institution ranking) who has participated in 19 papers. Professor Witell is in second place (13 papers, Universities of Linköping and Karlstad). Professors Vargo (University of Hawaiî at Mānoa) and Füller (University of Innsbruck) occupy the third and fourth places, authoring 12 documents each, although Vargo is the most cited author in the table followed by professors Lusch and Füller.

**TABLE 5:** Main researchers publishing co-creation related documents.

Author (Institution, Country)	Documents	Citations in WoS (of the documents) (20th May, 2020)
Edvardsson, Bo (Karlstad University, Sweden)	19	781
Witell, Lars (Linköping University and Karlstad University, Sweden)	13	533
Vargo, Stephen L (University of Hawai‘i at Mânoa, United States)	12	1599
Füller Johann (University of Innsbruck, Austria)	12	1109
Mahr, Dominik (Maastricht University, Netherlands)	10	406
Matzler, Kurt (University of Innsbruck, Austria)	10	719
Patricio, Lia (University of Porto, Portugal)	9	789
Maglio, Paul (University of California at Merced, United States)	9	970
Spohrer, James (‘Jim’) C (IBM, United States)	8	924
Lusch, Robert F (Arizona University, United States)	8	1277
Parida, Vinit (Luleå University of Technology, Sweden)	8	89
Pitelis, Christos (University of Leeds, England)	8	311
Reynoso, Javier (Tecnológico de Monterrey, Mexico)	8	237
Kristensson, Per (Karlstad University, Sweden)	8	666
Gustafsson, Anders (BI Norwegian Business School, Norway)	8	516
Skalen, Per (Karlstad University, Sweden)	7	500
Lievens, Annouk (University of Antwerp, Belgium)	7	340
McColl-Kennedy, Janet R (University of Queensland, Australia)	7	163
Jaakkola, Elina (University of Turku, Finland)	7	226
Breidbach, Christoph F (University of Queensland, Australia)	7	217
Buhalis, Dimitrios (Bournemouth University, England)	7	406
Sigala, Marianna (University of South Australia, Australia)	6	222
Zhang, Tingting (University of Central Florida, United States)	6	124
Sjödin, David (Luleå University of Technology, Sweden)	6	53
Trischler, Jakob (Karlstad University, Sweden)	6	64
Roberts, Deborah L (University of Nottingham, England)	6	152
Morosan, Cristian (University of Houston, United States)	6	78
Mele, Cristina (University of Naples Federico II, Italia)	6	119
Frantzeskaki, Niki (Swinburne University of Technology, Australia)	6	333
Hutter, Katja (University of Salzburg, Austria)	6	352
Kowalkowski, Christian (Linköping University, Sweden)	6	70
Dey, Bidit L (Brunel University, England)	6	45
Fisk, Raymond P (Texas State University, United States)	6	318

Table 6 gathers the collaboration statistics, considering authors, countries, and institutions as units of analysis. Focusing on authors, the most frequent kind of collaboration is among two or three researchers. In the last period, we observe noticeable growth in papers signed by three or more authors whilst the number of documents with two or fewer authors has gone down. In relation to countries, the most frequent case is when all the researchers are in one country, although we identify a positive evolution of international cooperation in the topic. Finally, we observe that the most frequent cases with respect to institutions collaborating is one or two. However, the number of cases with two or more institutions collaborating has grown in recent years.

**TABLE 6:** Collaboration in co-creation literature.

	Last period		Full period	
	2015–2020		2004–2020	
Authors	Documents	%	Documents	%
1	161	12.1%	227	13.3%
2	322	24.2%	457	26.8%
3	413	31.1%	518	30.3%
4	251	18.9%	296	17.3%
5 or more	182	13.7%	210	12.3%

**Countries**	**Documents**	**%**	**Documents**	**%**

1	797	58.9%	1050	60.7%
2	372	27.5%	461	26.6%
3	100	7.4%	123	7.1%
4	30	2.2%	36	2.1%
5 or more	27	2.0%	30	1.7%

**Institutions**	**Documents**	**%**	**Documents**	**%**

1	442	31.3%	604	33.6%
2	453	32.1%	589	32.7%
3	253	17.9%	304	16.9%
4	93	6.6%	104	5.8%
5 or more	85	6.0%	99	5.5%

### Bibliographic Coupling

**FIGURE 3:**
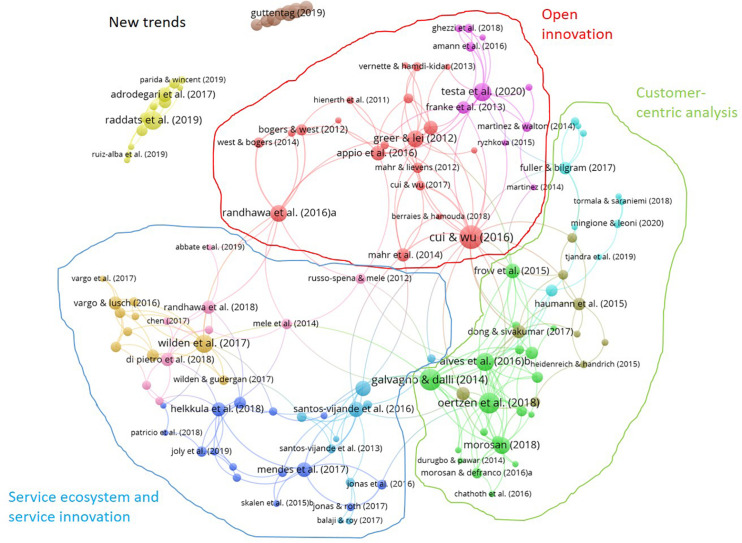
Bibliographic coupling network.

Figure 3 represents the network resulting from the bibliographic coupling analysis of 131 documents, following the procedure that we explained previously. As a result, we identify 11 clusters that we have classified in three research streams: (1) Open innovation (Table 7), (2) Customer-centric analysis (Table 8), and (3) Service ecosystem and service innovation (Table 9). This taxonomy derives from two sources. First, the relationship and closeness among topics dealt with by each cluster. We have studied all the documents included in the cluster, assigning a name to every cluster and analyzing the overlaps among them. These names appear in Tables 7–10, in which we summed up these clusters, along with some measures that complete their interpretation and the color identifying them in the network. The main topics studied in each cluster appear in the last column. Second, network analysis, which we used to confirm this taxonomy. To do that, we shrank the full bibliographic coupling network, transforming each cluster into just one node, which allows us to measure the intensity of relationships among clusters. Moreover, we used the VOS clustering algorithm to confirm the structure of our proposed research streams. Two of these clusters are disconnected from the main component. We refer to these clusters as new trends in technology and innovation management-related value co-creation literature, based on their composition (Table 10).

**TABLE 7:** Research stream 1: open innovation.

Cluster’s name	# docs	Year range/most freq. year/Av. year	# cits. per doc	h ind	Dens/centr	Journal specialization	Main topics
Innovation process and value co-creation (red)	20	2010–2019/2012/2014.35	99.40	15	80.30/1310	TIM (60.0%)	Consumer co-creation in new product development. Customer co-creation and open innovation. User innovation and co-creation.
Crowdsourcing, online communities and open and user innovation (pink)	10	2013–2020/2014/2015.80	21.30	7	60.20/479	Management (30.0%)	Open and user innovation. Crowdsourcing. Online communities. Social media-based innovation. Consumer value creation. Creativity.

**TABLE 8:** Research stream 2: consumer-centric analysis.

Cluster’s name	# docs	Year range/most freq. year/Av. year	# cits. per doc	h ind	Dens/centr	Journal specialization	Main topics
Theoretical issues about value co-creation (green)	19	2014–2020/2016/2016.53	36.05	13	94.63/1271	Management (21.1%); marketing (21.1%)	Definitions, theories and frameworks about value co-creation. Service-dominant logic paradigm. Theory of value co-creation. Perspectives and contexts of co-creation of value in business and management. Co-creation via the internet.
Brand and virtual value co-creation (light blue)	10	2010–2020/2011/2015.60	57.00	5	42.80/336	Marketing (70.0%)	Corporate brand management. Brand value co-creation. Co-creation experience. Multi-stakeholder perspective to brand co-creation. Virtual co-creation.
Co-created services/products and customer satisfaction (gray)	9	2012–2020/2015/2016.22	46.44	7	50.67/504	Marketing (44.4%)	Co-production. Customer participation. Customer co-creation. Customer satisfaction. Tourism services.

**TABLE 9:** Research stream 3: service ecosystems and service innovation.

Cluster’s name	# docs	Year range/most freq. year/av. year	# cits. per doc	h ind	Dens/centr	Journal specialization	Main topics
Value co-creation in service innovation ecosystems (marine blue)	15	2015–2019/2017/2016.80	24.47	9	54.00/662	Service (73.3%)	Service innovation ecosystem. Service design. Stakeholders in service innovation upon the co-creative paradigm. Value co-creation factors in service innovation.
Innovation, customer participation and performance (medium blue)	10	2012–2019/2013/2016.00	35.50	8	56.40/499	Marketing (70.0%)	Firm’s innovation capabilities. Ability to create value (performance and co-creation). Ability to create value for customer. Firm’s performance. Employees’ collaboration co-creation. Experience co-creation.
Service-dominant logic and value co-creation (orange)	10	2016–2018/2017/2016.90	89.70	9	78.40/586	Service (40.0%)	Service-dominant logic. Strategic approaches for service-dominant logic. Actors and service ecosystem. Coordination and cooperation involved in the co-creation of value. Business model.
Service innovation/value innovation (light pink)	9	2012–2019/2018/2016.22	58.67	6	43.56/504	Service (55.6%)	Service innovation. Open service innovation. Value innovation. Open/co-created process. Open innovation and knowledge co-creation. Digital service platforms.

**TABLE 10:** New trends in technology and innovation management-related value co-creation literature.

Cluster’s name	# docs	Year range/most freq. year/av. year	# cits. per doc	h ind	Dens/centr	Journal specialization	Main topics
Servitization (yellow)	12	2015–2020/2019/2017.83	13.58	8	89.00/n.a.	Management (50.0%); marketing (50.0%)	Servitization. Productization. Business model. Value co-creation capability.
Sharing economy and tourism industry (brown)	7	2019–2020/2019/2019.29	6.86	4	148.57/n.a.	Tourism (100.0%)	Sharing economy model. Hospitality and tourism management. Innovations in the tourism sector. Peer-to-peer (P2P). Airbnb

To analyze the clusters obtained from bibliographic coupling, we calculated different metrics about cluster size (number of documents), date (range of years, most frequent year, and average year of publication), and the scientific impact of the documents (citations per document and *h* index). We worked out the density of the cluster (the average degree of the cluster) to measure the intensity of the relationships among documents included in it, and the centrality (the weighted degree of each cluster in the entire network) to measure the relationship among clusters. We have classified the publishing journals in management, marketing, services, TIM, tourism, information technology (IT), and others, according to their aim and scope and we have identified the main topics in each cluster.

## Discussion

We have organized this section around the three research streams that we describe in the network (Figure 3): (1) Open innovation, (2) Customer-centric analysis, and (3) Service ecosystem and service innovation. The first one includes only two clusters, dealing with topics centered on the innovation process. The second research stream, which contains three clusters, analyzes topics related to the role of clients in the innovation. The third line, with four clusters, deals with themes focused on services industries and service innovation. Although all of them are closely related, for the sake of clarity, we analyze them independently. We have included a sub-section to analyze the emerging themes and another for the comparison of our results with previous literature.

### Research Stream 1: Open Innovation

The first research stream, Open innovation (Table 7), has received a more intense influence from the technology and innovation management literature. This research stream is present in other studies of a similar nature (Alves et al., 2016; Greenhalgh et al., 2016), although our study is closer to the innovation field. We find two clusters in this stream: the red one (Innovation process and value co-creation) and the pink one (Crowdsourcing, online communities and open and user innovation). They are located in the top zone of the network (Figure 3).

#### Innovation Process and Value Co-creation (Red Cluster)

In the red cluster, we can observe the different perspectives from which value co-creation concerning innovation is being studied: marketing, -e.g., service-dominant logic-, organizational behavior, -e.g., communities of practice-, and management, -e.g., dynamic capabilities- (Randhawa et al., 2016), and IT (Greer and Lei, 2012). In these studies, we have found two different levels of analysis: individual customers/users and customers/users associated with a company (Greer and Lei, 2012). An interesting topic included in this group is the study of customer co-creation during the innovation process as a major source for firms’ competitive advantage (Mahr et al., 2014). In this stream, we find concepts very similar to value co-creation: network collaboration, community innovation (West and Bogers, 2014), the creation of innovation-related knowledge in virtual communities (Mahr and Lievens, 2012) and the innovative brand community (Parmentier, 2015).

The red cluster is the oldest in the network. This fact explains, at least partially, why it is the group with the best performance in impact metrics (*h* index and citations per document). The inclusion of several literature reviews justifies the high number of citations and the variability among documents. Its density, one of the biggest, implies the strong connection among the documents included in the group. This is coherent with the homogeneity of the journals that have published these documents: 60% of the group has been edited by a TIM journal. This is the most central group, coherent with the nature of our database. In this sense, although a lot of these documents are published in journals of different specialties (management, service, marketing, and among others), it seems logical that all the papers in our database cite (with more or less frequency), seminal papers in this literature, most of them published in TIM journals. Finally, the average publication year does not imply, in this case, a process of abandoning this research line, because even though there are several papers published at the beginning of the analyzed period (Hoyer et al., 2010; Greer and Lei, 2012; Mahr and Lievens, 2012), there are some papers that have been edited recently (Cui and Wu, 2017; Morgan et al., 2018, 2019).

This cluster includes 20 documents (it is the biggest one) that deal with two main topics. Some of them focus on the involvement of customers in the new product development process (Hoyer et al., 2010; Greer and Lei, 2012; Mahr and Lievens, 2012; Cui and Wu, 2017; Morgan et al., 2018, 2019). The other subgroup of documents starts from the open innovation paradigm, approaching ideas like open service innovation (Randhawa et al., 2016), entrepreneurial orientation, market orientation, and resource orientation (Cheng and Huizingh, 2014), and user-centric value creation processes (Hienerth et al., 2011).

#### Crowdsourcing, Online Communities and Open and User Innovation (Pink Cluster)

The pink cluster includes papers that analyze open and user innovation (Amann et al., 2016), online communities (Testa et al., 2020), and crowdsourcing (Franke et al., 2013; García Martínez and Walton, 2014; Faullant and Dolfus, 2017; Ghezzi et al., 2018). The works in this cluster highlight the role of the consumer in the innovation process (García Martínez, 2014), especially the importance of the user’s or client’s creativity for innovation (Testa et al., 2020). Franke et al. (2013) comment that thanks to the Internet, new organizational forms have been created to integrate users into business innovation. There is a growing interest in online communities as a channel of innovation for businesses (García Martínez, 2015; Amann et al., 2016) and as an important source of knowledge and new ideas (García Martínez and Walton, 2014). Ryzhkova (2015) confirms the importance of collaboration with customers, supported by information and communication technology (ICT), for the innovation performance of companies.

Crowdsourcing is the other big pole of attraction in this cluster. For Ghezzi et al. (2018), this phenomenon is rooted in two main disciplines within the broader theme of innovation and management: (1) open innovation; and (2) co-creation. Faullant and Dolfus (2017) commented that virtual crowdsourcing initiatives, and in particular crowdsourcing competitions, are a means of harnessing users’ creativity to aid corporate innovation. Crowdsourcing can be considered as a source of innovation and creativity (García Martínez, 2015) and as a tool for data analysis that helps to manage “Big Data” in companies (García Martínez and Walton, 2014).

This cluster is closely related to the “Innovation process and value co-creation” one, although its characteristics are completely different. It has a lower number of citations per document than other groups with similar average publication dates although its *h* index is better. Also, it is remarkable that in the last 2 years, just one paper included in this group has been published. All these data together point to this group having less potential than others in this literature. The density of this group implies a moderate connection between papers, which is coherent with the variable nature of the publications that have edited these documents. The most frequent journal category is management with 30% of documents, followed by TIM with 20%. Its centrality is also modest, with a strong connection only with the other cluster in this research stream.

### Research Stream 2: Customer-Centric Analysis

In this research stream (Table 8), we find three clusters that analyze topics related to different facets of the consumer/user: satisfaction, engagement, and participation, among others. These clusters are the green one (Theoretical issues about value co-creation), the gray one (Co-created services/product and customer satisfaction), and the light blue one (Brand and virtual value co-creation). This research stream occupies the right side of the network.

#### Theoretical Issues About Value Co-creation (Green Cluster)

The green cluster includes 19 documents that establish conceptualizations (Ramaswamy and Ozcan, 2018) and theoretical frameworks for this literature, in a general context (Martínez-Cañas et al., 2016; Chen et al., 2017), as well as in specific ones. For example, Oertzen et al. (2018) propose a model for services, while Chathoth et al. (2016) and Hamidi et al. (2020) focus on tourism. Some of them include a literature review (Baron et al., 2014; Bharti et al., 2015; Chathoth et al., 2016; Oertzen et al., 2018) and others use bibliometric techniques (Galvagno and Dalli, 2014; Alves et al., 2016). Although this cluster has a more intense link with the other clusters included in its research stream, it has connections with all the clusters, due to its more generic nature (in this field).

In this group, we find works that consider that value co-creation represents a critical element of the service-dominant logic paradigm (Baron et al., 2014; Morosan and DeFranco, 2016; Morosan, 2018). Other authors consider theories such as the theory of value co-creation (Galvagno and Dalli, 2014), the theory of service co-production, and some models of theory-building approach (Grace et al., 2019). Also, we find the work of Galvagno and Dalli (2014), which presents three perspectives of literature study on co-creation: service science, innovation and technology management, and marketing and consumer research.

Some works in this group highlight perspectives and contexts of the usage of the term value co-creation in business and management fields. Also, this group focuses on diverse approaches and areas that study co-creation. For example, Alves et al. (2016) consider value co-creation as a driver of business innovation, the development of new products and services, the experience of consumers of brands, and the co-creation process. In some cases, innovation appears in theoretical models as a perspective for the study of value co-creation or as a factor (Durugbo and Pawar, 2014; Jouny-Rivier et al., 2017). Among these works, we highlight the study of Bharti et al. (2015) that identifies 27 elements of co-creation classified into five categories: process environment, resource, co-production, perceived benefits, and management structure.

Papers in this cluster have an average of 36.05 citations. Most of the documents are relatively recent (in fact, all of them were published after 2014), which explains the low impact metrics, at least partially. However, it is remarkable that although the number of citations per document is much lower than other clusters (for example, the red cluster), its *h* index is 13. This means there is less variability in the impact of documents that form the cluster. With one exception, this group has the highest density, which implies the biggest number of shared references among papers in the cluster, thus the biggest proximity among topics. However, this fact contrasts with the variability in the specialty of the journals that have published these documents: the two biggest groups are management and marketing, with 21.1% of the documents. Service and tourism are also important categories in this group. This diversity explains the high centrality of the cluster, the second-highest overall.

#### Co-created Services/Products and Customer Satisfaction (Gray Cluster)

In close proximity to the green cluster, the documents that comprise the gray cluster deal with topics related to customer satisfaction (Grissemann and Stokburger-Sauer, 2012; Haumann et al., 2015; Heidenreich et al., 2015). Most of the articles in this group analyze co-creation in the service context, with special attention to the tourism industry and technology-based services (Heidenreich and Handrich, 2015; Stokburger-Sauer et al., 2016; Dong and Sivakumar, 2017; Sarmah et al., 2017; Kamboj and Gupta, 2020).

Dong and Sivakumar (2017) analyze customer participation, customer commitment and customer innovation as related but distinct concepts. Starting from a vision of customer participation in services, Blinda et al. (2019) classify the characteristics of the participation process as experience-oriented versus results-oriented. Kamboj and Gupta (2020) mainly examine the customer perspective on service innovation. Authors such as Heidenreich and Handrich (2015), Sarmah et al. (2017), and Kamboj and Gupta (2020) consider the basic technology-based service adoption model applied to the tourism sector and study the impact of innovation, the willingness to co-create, the need for interaction and its effect on results (adoption intent and customer satisfaction).

Another term that appears in this group is co-production, which is considered a component of value creation, offering many benefits for customers and management, but also requiring customers to invest a considerable amount of time and effort (Stokburger-Sauer et al., 2016). Haumann et al. (2015) comment that little is known about the strategies that companies can employ to positively influence customer perceptions of co-production processes.

This cluster has a moderate number of citations per document but a better *h* index that implies a more homogenous impact of the papers included in it. The documents of this cluster are published by a blend of marketing, service, and tourism journals, as a consequence of a variety of topics. Its low-density figure points to a moderate connection among the documents. The moderate centrality also implies a low-intensity link with other groups. Although its average publication year is 2016.22, there is a high dispersion of articles along the considered period. We have to highlight that this group includes two papers published very recently (Blinda et al., 2019; Kamboj and Gupta, 2020).

#### Brand and Virtual Value Co-creation (Light Blue Cluster)

The light blue cluster has two subgroups: one group around the research of Füller and Bilgram (2017) and another group around the work of Ramaswamy and Ozcan (2016). The first subgroup analyzes virtual co-creation platforms and the co-creation experience (Füller et al., 2011; Kohler et al., 2011; Füller and Bilgram, 2017). This subgroup is very close to the first research stream, as we can see in the network, sharing some topics. The other subgroup focuses on brand value co-creation (Hsieh and Chang, 2016; Ramaswamy and Ozcan, 2016; Tormala and Saraniemi, 2018; Mingione and Leoni, 2020) and corporate brand management (Schmidt and Redler, 2018). This cluster considers specifically the role of other stakeholders in the co-creation process.

Digging deeper in the first subgroup, around the central concept of virtual co-creation, Füller et al. (2011) analyze ‘virtual design skills’ as a means to develop the innovation process and enrich companies. Kohler et al. (2011) emphasize the importance of experience in fostering active participation in innovation tasks. Virtual co-creation is considered a viable strategy for developing consumer-centered products in the digital age (Füller and Bilgram, 2017).

In the other part of this cluster, Ramaswamy and Ozcan (2016) present an integrative framework for brand value co-creation. They describe how brand engagement platforms work, their different functions and roles, and how they connect companies to stakeholders. Hsieh and Chang (2016) propose an integrative theoretical framework to synthesize perceived psychological benefits and distinctive motivations in the brand co-creation process. Schmidt and Redler (2018) analyze corporate brand management from a strategic perspective. Tormala and Saraniemi (2018), Tjandra et al. (2019), and Mingione and Leoni (2020) provide a multi-stakeholder perspective for brand co-creation and analyze the co-creative actions of the corporate brand.

This cluster contains three papers from 2010 and 2011 with the rest of the documents published in 2016 or after. This bipolarity explains its low density (the lowest). Also, the cluster has a moderated centrality, with strong connections to the green cluster (“Theoretical issues about value co-creation”) especially with the group around Ramaswamy and Ozcan’s (2016) work, and to the red and pink clusters (more focused in TIM), through Füller and Bilgram’s (2017) research. This division is also noticeable in the journals that have published the documents, with a bigger group concentrated in marketing related publications, and the other without a clear specialty. Although 70% of the documents in this group are in marketing-related journals, this trend is more noticeable in the subgroup that deals with brand value co-creation. We can observe a high dispersion in several characteristics: the year of publication that ranges from 2010 to 2020 and a higher number of citations per document but a smaller *h* index, which implies that a significant proportion of citations are concentrated in just a few of the papers in the group.

### Research Stream 3: Service Ecosystems and Service Innovation

On the left side of the network, we find four clusters that constitute the third research stream (Table 9): service ecosystems and service innovation. We have called these clusters “Value co-creation in service innovation ecosystems” (marine blue), “Innovation, customer participation and performance” (medium blue), “Service-dominant logic and value co-creation” (orange), and “Service innovation/value innovation” (light pink). All of them deal with topics related to value co-creation in services, analyzing themes like service design, stakeholders in service innovation, or service innovation ecosystems.

#### Value Co-creation in Service Innovation Ecosystems (Marine Blue Cluster)

All the documents in the marine blue cluster deal with different facets of service innovation. In the group, we find several conceptual studies (Skalen et al., 2015; Foglieni and Holmlid, 2017; Mendes et al., 2017; Helkkula et al., 2018; Yu and Sangiorgi, 2018; Joly et al., 2019), some case studies that analyze the role of different agents in service innovation (Karlsson and Skalen, 2015; Chew, 2016; Jonas et al., 2016), and works that explore service innovation in the health industry (Beirao et al., 2017; Jonas and Roth, 2017; Patricio et al., 2018).

This group of studies highlights the different fields that have contributed to the analysis of customer value co-creation in service innovation (Jaakkola et al., 2015; Chew, 2016; Joly et al., 2019). Starting from this multidisciplinarity, but always focused on service innovation, the documents included in the cluster have analyzed different issues: service experience co-creation (Jaakkola et al., 2015), the relationship between value co-creation and service evaluation (Foglieni and Holmlid, 2017), and the role of different stakeholders (Karlsson and Skalen, 2015; Akesson et al., 2016; Jonas et al., 2016; Jonas and Roth, 2017).

Yu and Sangiorgi (2018) state that “Service design, as a human-centered and creative approach to service innovation, can reframe new service development processes to implement value co-creation.” This is evident in healthcare services (Beirao et al., 2017; Patricio et al., 2018) and medical appliances (Jonas and Roth, 2017).

This cluster is the biggest in this research stream, contains 15 papers, and has a clear specialization in service-related publications (73.3% of documents were published in journals related to this topic). This is the most central cluster in this research stream, which is consistent with the more conceptual nature of several documents it includes. Its combination of high *h* index (9) with low citations per document (the lowest in the group) is due to some of the studies being very recent while others have been highly cited in this context.

#### Innovation, Customer Participation, and Performance (Medium Blue Cluster)

The medium blue cluster contains several documents analyzing different issues that link co-creation with performance (O’Cass and Ngo, 2012; Zhang et al., 2015; Santos-Vijande et al., 2016; Anning-Dorson, 2018). Some of them also focus on innovation (Ngo and O’Cass, 2013; Santos-Vijande et al., 2013; Kautish and Sharma, 2019). This diversity found a shared point in the service industry, with 80% of the papers analyzing it.

Santos-Vijande et al. (2013) investigate the relationship between innovative culture, innovation efforts and performance in knowledge-intensive business services (KIBS). Santos-Vijande et al. (2016) note that from a service-dominant logic perspective, employees are operational resources that companies can consider to improve innovation results. Anning-Dorson (2018) analyzes the influence that customer participation capacity has on the performance of service companies and considers that innovation has a mediating effect on this relationship. O’Cass and Ngo (2012) and Ngo and O’Cass (2013) study the effect of innovation and client engagement on performance and competitive advantage.

This cluster has a consistent evolution during the period, with papers published from 2012 to 2019. Also, even though the number of citations per document is not very high, its *h* index is remarkable, which implies less variability in the citations among papers. There is a clear specialty, with 7 of the 10 papers forming the cluster published in marketing-related journals, although most of them deal with service industries. The moderated density points to a weaker relationship among papers in the cluster as the medium centrality implies there are not intense connections with other groups, although some papers, specifically Santos-Vijande et al. (2016, 2018) are connected with several clusters. The consistency of the group suggests a good potential for development.

#### Service-Dominant Logic (Orange Cluster)

Most of the documents in the orange cluster focus on this topic. Among all the works that make up this group, Vargo and Lusch’s (2016, 2017) research stands out, introducing the concept of “service ecosystems.” These theoretical documents try to consolidate the bases for the development of this theory in the marketing area.

Service-dominant logic emerged more than a decade ago as a potential framework for addressing the role of service in exchange and value creation (Vargo and Lusch, 2017; Wilden et al., 2017). From this perspective, the absence of coordination and cooperation mechanisms involved in value creation can be observed, but the concept of the service ecosystem can be included as a new axiom, focusing on the role of institutions and institutional arrangements in value creation systems (Siltaloppi et al., 2016; Vargo and Lusch, 2016). Polese et al. (2018) present an integrated model, the so-called intelligent service ecosystem that can be applied to hyper-competitive and experience-based sectors and that explores the main elements-steps to manage and optimize value co-creation and long-term sustainability and, therefore, to move from innovation to social innovation.

This cluster has high density (which implies a strong connection among its components) and moderate centrality (with just a few connections to other clusters). The impact metrics are notable, in the number of citations per document as well as in the *H* index. Also, although this group is relatively young (2016.8), we have to highlight the concentration of all the documents included in the cluster in 3 years: from 2016 to 2018. The kind of publication that has edited the papers is a mix of service and marketing. The presence of professors Vargo and Lusch (Lusch et al., 2016; Vargo and Lusch, 2016, 2017) in several of these documents is also significant, although the research of professor Wilden (Ralf and Siegfried, 2017; Wilden et al., 2017) is the main link of this group with others, especially with the marine blue cluster.

#### Service Innovation/Value Innovation (Light Pink Cluster)

We refer to the light pink cluster as “Service innovation/value innovation” because most of the documents revolve around these topics (Mele et al., 2014; Lusch and Nambisan, 2015; Koskela-Huotari et al., 2016; Chen, 2017; Wallin and Fuglsang, 2017; Di Pietro et al., 2018; Randhawa et al., 2018). Russo-Spena and Mele (2012) approach innovation as a co-creation process from a practice-based vision and develop the five “Co-s” model. In a similar line, Mele et al. (2014) offer a framework for innovation. It is based on the comparison of the three research approaches: (1) goods-dominant logic (development of new products and services and the company as the main actor); (2) resource-based approach (innovation drivers such as knowledge, skills and relationships); (3) service-dominant logic (“open” innovation processes in which all network actors can mobilize and integrate their resources to become value co-innovators).

Lusch and Nambisan (2015) develop a framework of analysis for service innovation based on three pillars: (1) service ecosystems; (2) service platforms; and (3) value creation. Chen (2017) analyses service innovation in different service providers and, from the service-dominant logic perspective, presents four models of service innovation development: ICT platforms, customer relationship management systems, community trading services and multi-channel services. Randhawa et al. (2018) examine how intermediaries in general, and those with digital service platforms specifically, engage with customers to help them innovate their services within their service ecosystem. Abbate et al. (2019) analyze how open innovation digital platforms function as “co-creating intermediaries” that define, develop and implement dedicated processes, specific tools, and appropriate services to support knowledge co-creation activities.

The concentration of documents in service-related journals (55.6%) reflects a more focused approach to service topics. The cluster density points to a weaker connection among the components of the cluster. Also, there is a higher dispersion in the number of citations, as we can observe in citations per document and *h* index figures. The moderate centrality also implies a low-intensity link with other groups, although we can observe some relationship to the orange (“Service-determinant logic”) and blue marine clusters. Its average publication year hides that papers have been published throughout the period. Some recently published documents point to the vitality of the topic (Di Pietro et al., 2018; Randhawa et al., 2018; Abbate et al., 2019).

### New Trends in Technology and Innovation Management-Related Value Co-creation Literature (Yellow and Brown Clusters)

Finally, the yellow and brown clusters are separated from the network, which implies that their relationship with the rest of the clusters is weaker (Table 10). In this situation, calculating centrality does not make sense. Moreover, they are the most recent groups. We have named the yellow cluster “Servitization” because all the documents this group contains deal with this topic. We have found several conceptual papers (Huikkola and Kohtamäki, 2017; Palo et al., 2019; Raddats et al., 2019; Ruiz-Alba et al., 2019) and some case studies (Windahl, 2015; Windler et al., 2017) that contribute to set the fundamentals of this literature. This cluster has a high density, which implies that papers comprising it have a strong connection. The twelve papers that form this cluster are published only in journals specialized in management and marketing. This literature shows a high-potential of growing in the next several years.

Several of the works in this group conceptualize the term servitization. Story et al. (2017) note that “servitization involves manufacturers developing service offerings to increase revenue and profits and include customers, co-creating innovation as advanced service capabilities for each player.” Raddats et al. (2019) explain that “servitization describes the addition of services to manufacturers’ core product offerings to create additional value for the customer.” Also, Palo et al. (2019) extend the “conceptualization of servitization as a bottom-up, emergent and iterative process of business model contestation.” One of the most recent works introduces the analysis of productization (Li et al., 2020) into this group.

With a more specific focus, Parida and Wincent (2019) relate the concepts of sustainability, business models, innovation, and networks to examine new trends in digitization, the circular economy, and servitization. Ruiz-Alba et al. (2019) study, from the client’s perspective, the moderating role of co-creation in the implementation of servitization strategies and its effects on performance.

The brown cluster is completely specialized in tourism, with 100% of papers published in journals related to this topic. We have called it “Sharing economy and tourism industry.” Some of these works focus on the sharing economy and peer-to-peer (Altinay and Taheri, 2019; Tang et al., 2019; Belarmino and Koh, 2020). Airbnb is the case study for some papers in this cluster (Dann et al., 2019; Guttentag, 2019; Zhang et al., 2019). All the papers in this group have been published in 2019 and 2020, which explains why the impact measures of this cluster are the lowest. The other characteristic that is remarkable in this group is that it is the densest one, which implies the strongest connection between the papers that are included in it. This cluster has a high potential for developing in the next several years.

In this group, value co-creation is considered as an element within the sharing economy. Hossain (2020) reviews the existing literature on the sharing economy and shows how sharing economy companies operate novel business models with unique revenue streams. This study points out the economic, social and environmental impacts of the sharing economy. Altinay and Taheri (2019) review and synthesize recent studies in the sharing economy literature and identify the knowledge gap and future opportunities for researchers, especially as applied to the tourism sector.

### Comparison With Previous Literature

Galvagno and Dalli (2014) pointed out that the TIM perspective was underdeveloped. Our study has focused on that perspective in the last 10 years. We present a map with the main research streams in this literature. Specifically, we find three research streams: “open innovation,” “consumer-centric analysis,” and “service ecosystem and service innovation.” Also, we observe two emerging trends that we have called “servitization” and “sharing economy.”

**FIGURE 4:**
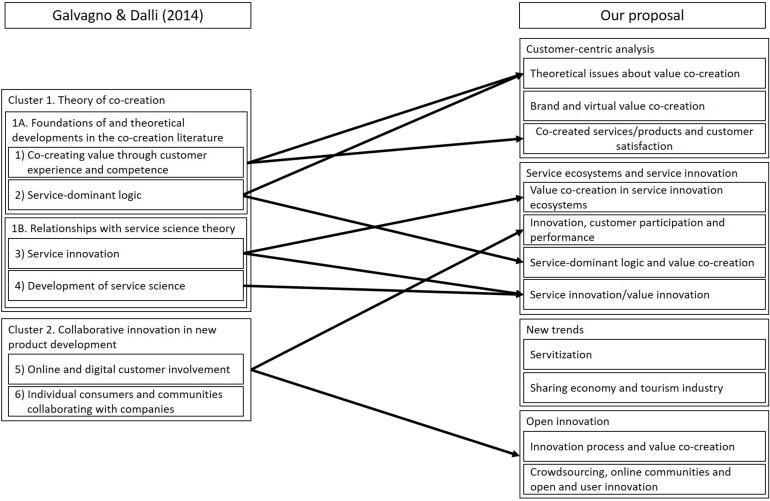
Relationships between Galvagno and Dalli’s knowledge structure and ours.

Although our focus is narrower than Galvagno and Dalli’s work, our results are comparable because technology and innovation are issues that permeate the value co-creation literature. In Figure 4, we have represented the main relationships between this model and ours. Although these results emerged from the analysis of the literature from 2000 till 2012, we found a strong correspondence between both knowledge structures. The main differences are due to the evolution of the topics. However, we can affirm that both structures shared several elements in common. Cluster 2 in Galvagno and Dalli’s proposal (collaborative innovation in new product development) has remained a research stream, although some of the topics are analyzed from the services perspective, the main field in value co-creation in recent years. Also, we observe a trend to blend issues. Service-dominant logic has become the most referenced framework in value co-creation, being one of the basic pillars in documents more related to marketing as well as in those more focused on services.

Another interesting issue emerges from the comparison of both models: although value co-creation is not an exclusive phenomenon in service industries, in recent years, the majority of studies have focused on this kind of industry. Public services, health-care, and especially tourism have gathered the attention and effort of researchers in value co-creation. We identify a cluster oriented completely to services and new trends that, in different ways, consider services as a main component.

Galvagno and Dalli (2014) observe two levels of analysis in co-creation studies: company and customer. We have found that in recent years, although some studies consider a company-level analysis, most of them have focused on a customer experience-centered perspective. This is noticeable in all the literature but especially in the “consumer-centric analysis” research stream and in the pink cluster “crowdsourcing, online communities and open and user innovation.” The “service ecosystems and service innovation” research stream is more heterogeneous, with some of the groups focused on companies’ performance and innovation systems. The red cluster “innovation process and value co-creation,” included in the “open innovation” research stream, also deals with this perspective.

## Conclusion

In the introduction, we laid out several research objectives. The first refers to the development and social structure of the value co-creation literature. The bibliometric analysis has revealed some interesting conclusions. The exponential growth in scientific production, especially in the last 5 years, confirms the interest of the academic community in the value co-creation phenomenon.

This scientific production has spread in publications addressing diverse themes. Initially, most of the research papers were published in management and business journals (according to WoS categories). Evolution has modified this trend, incorporating new and different publications. The analysis shows the importance of marketing and TIM journals. However, in recent years, the services field is the biggest pole of attraction for this literature.

European universities have stood out. Several of the most prolific authors (Edvarsson, Witell, Vargo, and Füller) are working for European universities and, in particular, for institutions from Netherlands, Nordic countries, England, and Central Europe. Karlstad University, Linköping University, and Innsbruck University have very active teams working on this topic.

Regarding collaboration, we observed a noticeable growth in papers signed by three or more authors in the last 5 years. This fact fits with the topic’s evolution toward maturity. The affiliations of co-authors also reflect this cooperation. Association among researchers of the same country is the most frequent situation, but documents involving researchers from different nations are habitual.

To complete the second goal, we have mapped the structure of knowledge of value co-creation in the TIM field. We have delimited three research streams, which are the backbone of the knowledge structure of value co-creation in the TIM context: Open innovation, Customer-centric analysis, and Service ecosystem and service innovation. We have found strong links among them, and specifically among the clusters that form them.

The first research stream, Open innovation, includes two thematic clusters that deal with topics like innovation process, crowdsourcing, and online communities and their role in user innovation. The documents with the most significant influence in the TIM field are in this line. Open innovation and new product development have been central themes, and more specific issues have appeared in recent years. Several papers in this stream constitute a basic pillar of this literature.

The Customer-centric analysis research stream deals with value co-creation from the marketing perspective. Innovation is present but combined with specific theories of value co-creation. Customer satisfaction, participation, corporate brand management, or co-production experience are some of the topics that have developed in this line.

The third research stream, Service ecosystems and service innovation, includes the papers that analyze value co-creation from the service perspective. In this stream, the Service-dominant logic paradigm acquires a superior dimension, becoming a central theory in this field. Service innovation is the main studied concept. Aspects such as the process, the actors, the capabilities to create value, or the service design, among others, form this line, the most active of the three. The growing maturity of the topic has driven it to analyze more specialized issues. The comparison with previous knowledge structures in this field shows that the main research streams remain but with more and more specialized groups inside of them.

From a general point of view, the customer retains the central role in value co-creation. Thus the marketing field is one of the main poles of attraction for researchers in this concept. However, two circumstances have modified this panorama in recent years: first, the growing interest of researchers in services in this topic. Considering that services and marketing are complementary issues, we have to highlight the higher intensity of the presence of researchers, publications, and issues related to service industries. One of the research streams focuses on service issues. Second, the introduction of other relevant agents in co-creation literature is more common in recent years, especially for employees.

The analysis has let us identify some emerging topics and some future research lines. In the first research stream, Open innovation, we have to highlight the research of the involvement of customers in new product development. This research is incorporating new measures for process performance or involvement/participation, and considering more complex variables and models, like the type of customers, the optimum level of involvement, or search and coordination costs of the process. In a more specific context inside this research stream, the research of Ghezzi et al. (2018) in crowdsourcing and the work of Testa et al. (2020) in social media-based innovation identify several research gaps that will focus the effort in this topics in coming years.

In the Customer-centric analysis research stream, we have found some topics that are gaining interest. We have to highlight the issues around corporate brand value co-creation. Also, the analysis of the role of intermediaries in the value co-creation process, connecting customers with companies, is another specific theme that is growing in the field. Other ways to get this participation and the implications of use (e.g., apps) have to be explored further in the future.

The Service ecosystems and service innovation research stream has been the most active in recent years. Several works have pointed to some of the main topics to analyze in the coming years. Service-dominant logic has played an essential role in this field and remains one of the poles of attraction for this literature. Vargo and Lusch (2017) have done an excellent job organizing these works and establishing future research lines. Service design is another leading topic in this stream. Helkkula et al. (2018) suggest a future research agenda around service innovation.

Two emerging themes join these research lines: servitization and sharing economies. Academics have focused on these topics in the last several years. Raddats et al. (2019) and Hossain (2020) outline comprehensive reflections about the evolution of these themes. All these conclusions reinforce the growing prominence of the service field in the value co-creation arena.

From a theoretical point of view, our study offers three principal contributions. First, we have given a dynamic image of the field in recent years, describing the activity in it, characterizing the most usual journals publishing this literature, and gathering the main actors working in it (authors, institutions, and countries). All of it assesses the importance of the topic and maps its social structure.

Second, we have studied the different topics in this literature, describing its knowledge structure. For each thematic group, we have characterized its role in the literature and its evolution. We have compared our results with previous studies to figure out how some themes have remained over time while others have disappeared.

Third, we have delimited two emerging themes and several future lines for each research stream. The academic community should consider our proposals for addressing in depth the joint analysis of value co-creation and TIM. On the other hand, our study should be useful for researchers focused on the service industries, such as tourism, public services, or health care.

From a practical perspective, our study has another three implications. First, this paper assesses the dimension of value co-creation in the business context. This phenomenon has become a competitive necessity in some industries. Co-creation is essential to deliver services that day by day are growing, not just in service-related industries. Managers should consider value co-creation as a priority on their agenda.

Second, this literature highlights the role of the customer from a more general point of view. Clients have assumed new roles. Now, they are innovators, information sources, co-producers, communicators, and even more. Companies have to learn what kind of customers could play those roles, how to manage each one of these functions, and how much participation is optimum for the company. Managers have to respond to all of these questions as soon as possible.

Third, we have identified the service industry as one of the most relevant in the value co-creation process. In tourism, public, and health care services industries, the value co-creation has grown exponentially in the last few years. Interaction with customers in the design or production of services has become essential for companies in these industries. Our study has shown some of the questions that these companies have to address.

Finally, this study is not free of limitations. As we mentioned in the methodological section, the use of the bibliographic coupling technique implies decisions that influence the final result. Although we have checked that our sample is representative, it could be improved. Also, we can use co-citation methods to analyze the intellectual structure of the field. However, space limitations require us to leave this task as a future research line for this work. More specific and detailed analyses are, also, future research possibilities.

## Data Availability Statement

All datasets generated for this study are included in the article/Supplementary Material.

## Author Contributions

All authors listed have made a substantial, direct and intellectual contribution to the work, and approved it for publication.

## Conflict of Interest

The authors declare that the research was conducted in the absence of any commercial or financial relationships that could be construed as a potential conflict of interest.

## Supplementary Material

The file can be open with Bibexcel.
